# Editorial: Nanosized Drug Delivery Systems: Colloids and Gels for Site Specific Targeting

**DOI:** 10.3389/fbioe.2020.00803

**Published:** 2020-08-04

**Authors:** Martin Ehrbar, Filippo Rossi, Francesco Cellesi

**Affiliations:** ^1^Department of Obstetrics, University Hospital Zurich, University of Zurich, Zurich, Switzerland; ^2^Department of Chemistry, Materials and Chemical Engineering, Politecnico di Milano, Milan, Italy

**Keywords:** nanomedicine, drug delivery, nanocarrier (nanoparticle), colloids, gels, targeting, growth factors

Recent advances in nanomedicine and biomaterials have provided new promising tools for *in vitro* models and targeted drug administration *in vivo*, aiming to increase efficacy while limiting side effects. Nanosized drug delivery systems are designed to modify the biodistribution of therapeutic agents, in order to enhance their accumulation in the pathological site. Bioactive molecules can be either conjugated to or entrapped into colloidal nanocarriers. Biocompatible nanoparticles (NPs), polymer nanotherapeutics, lipid-based nanomaterials may enhance the stability and the targeted delivery of low molecular weight drugs, as well as nucleic acids and therapeutic proteins. Once administered *in vivo*, these colloids will face sequential biological barriers, which represent a major challenge for site specific drug delivery. A fine control of key physicochemical characteristics of the nanocarriers, including size, drug loading, and functionality, may lead to a successful barrier penetration and an effective release at the targeted site.

Thomas and Weber review current limitations of NP-based drug delivery by focusing on cancer treatments. While enhanced permeability and retention (EPR) can result in drug accumulation in malignant tissues, this effect is highly dependent on tumor type and location. In order to improve drug delivery, drugs, and NPs with long half-lives are obtained by conjugation or coating with hydrophilic polymers. Controlled activation approaches and active targeting to cancer cells or specific cellular compartments are discussed in view of possible future developments.

Naturally derived polymers are increasingly used in therapeutics and diagnostics. Chitosan NPs are known to be promising vehicles for drug, protein, and gene delivery. Their main drawback resides in their low physical and chemical stability under biological conditions. In order to overcome this limitation, Saeed et al. grafted chitosan to phthalic or phenylsuccinic acids and then used polyphosphoric acid, hexametaphosphate, or tripolyphosphate to achieve ionotropic complexation and covalent crosslinking by carbodiimide chemistry. The NPs showed high stability and reproducibility, as well as cytotoxic activity once loaded with the chemotherapic doxorubicin.

Vashist et al. developed a smart strategy to prepare auto-fluorescent hydrogel NPs made of hydroxyethyl cellulose and chitosan, using water-in-oil emulsion polymerization. These nanoparticles guaranteed biocompatibility, stability, proper cellular uptake, and the ability to cross a model of blood brain barrier. Moreover, their auto-fluorescence in a wide range of wavelengths may be exploited in the field of theranostics to develop image-guided therapies for the central nervous system.

Recently, polymeric NPs have been tested as therapeutic cancer vaccines, i.e., medical tools which are capable of educating the immune system to fight tumors and prevent cancer diseases. Briquez et al. discuss in their review the principles and current strategies to engineer therapeutic cancer vaccines, with a particular focus on the use of site-specific targeting nanomaterials. They outline the type of immune responses triggered by vaccination, with an overview of the main components of cancer vaccines, describing how nanomaterials can be engineered to improve pharmacokinetics and pharmacodynamics of the vaccine.

Current barriers to clinical translation of NPs are mainly related to the limited control over NP physicochemical properties and robust scale-up of their production. Bovone et al. engineered an automated coaxial jet mixer for the production of stable and size-controlled polymeric NPs from chemically diverse block copolymers. The jet mixer allowed a fine tuning of the flow conditions and mixing of the copolymer-containing organic phase and an aqueous stream, for the continuous nanoprecipitation of polymeric NPs.

The advantage of protecting active compounds from the environment during storage and transport, while achieving a safe and controlled release, are also relevant for applications which are far from healthcare. Vega-Vásquez et al. describe different types of nanocarriers developed in the biomedical field which may find applications in agriculture, specifically in the area of plant breeding, growth promotion, disease control, and post-harvest quality control, in order to improve plant and food production, while reducing the impact on the environment.

Moncalvo et al. describe in their review the recent research progress on nanosized delivery systems for therapeutic proteins, highlighting future directions and challenges. Although the impact of protein therapeutics in healthcare is steadily increasing, their safety and efficacy are often limited by instability, short half-life, and immunogenicity. Covalent attachment of biocompatible polymers, as well as protein nanoencapsulation in colloidal systems, are currently being investigated for overcoming these limitations, with the potential to develop next-generation protein therapeutics.

Smart hydrogels with highly ordered structures at the nano-scale have been extensively investigated as advanced delivery systems. These gels can act as efficient drug protectors, especially for peptides and proteins with limited half-life, and as targeted drug carriers for *in situ* controlled delivery. These characteristics are particularly appealing in tissue engineering, where extracellular matrix (ECM) mimetic biomaterials are required for controlled release of soluble factors to stimulate tissue regeneration.

Ren et al. review growth factor (GF) engineering strategies for tissue regeneration. GF binding to provisional biomaterials or even natural ECM serves as slow-delivery approach. The stability of GFs may be enhanced by site specific PEGylation, by the optimization of the primary structure or the modification of proteolytic sites. GF signaling may also be improved by modulating the receptor binding site, creating hybrid proteins which engage alternative signaling pathways, or controlling the GF availability to cell-surface receptors.

Hwang et al. review ECM-mimetic biomaterials for targeted and sustained drug release. The broad repertoire of ECM interactions with different cell-surface receptors can facilitate cell-targeted delivery and improve the therapeutic efficiency of drugs. In addition, the interaction between ECM components and cellular receptors are exploited for intracellular delivery, which may have a significant impact on disease treatment and tissue regeneration.

Fattah and Ranga present the latest advances in engineering organoids through a modular and orthogonal design of biomimetic materials, and the use of NPs which are able to modulate matrix and tissue mechanical stresses. Magnetic NPs enable the localized mechanical manipulation of cells and biomaterials during the establishment of organoids. Once internalized by target cells, these NPs become tools for cell stimulation and assembly of complex tissue structures.

## Author Contributions

All authors listed have made a substantial, direct and intellectual contribution to the work, and approved it for publication.

## Conflict of Interest

The authors declare that the research was conducted in the absence of any commercial or financial relationships that could be construed as a potential conflict of interest.

